# Supplementary material for: Two-dimensional reward evaluation in mice
Source: Anim Cogn. 2021 Mar 15;24(5):981–98. doi: 10.1007/s10071-021-01482-8 (PMC8360905; doi:10.1007/s10071-021-01482-8)

Electronic Supplementary Material

Figure S1. **Total number of nose pokes for each experimental condition in the three cohorts in all experiments.** Rows show different experiments (1-4). Each symbol represents the total number of nose pokes for a single mouse over one of the two experimental days of the given condition.

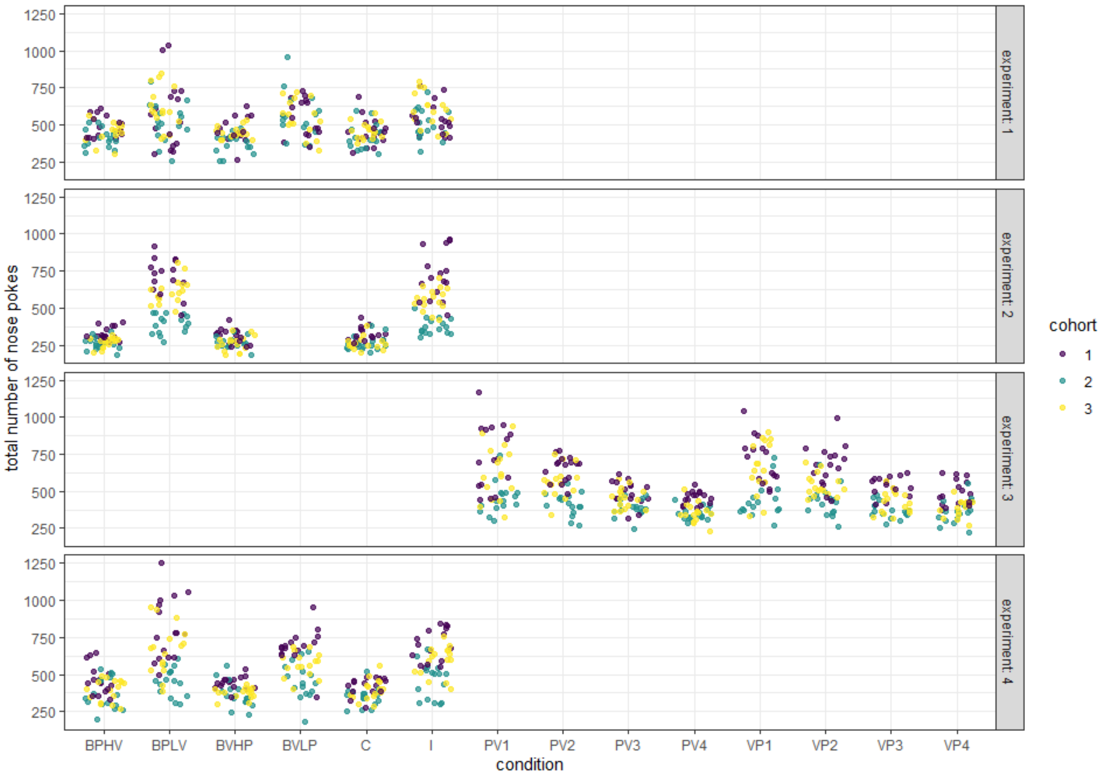

Figure S2. **Learning curves in experiment 1.** Only nose pokes at the rewarding dispensers were included (abscissa). Dots give the mean discrimination performance for blocks of 20 nose pokes over the respective number of mice (dot size) and the error bars give the standard errors. Purple symbols correspond to the first acquisition of a new condition (rows) and yellow symbols correspond to the reversal day of the same condition. The columns give the different cohort numbers (1-3). The horizontal dotted line corresponds to chance performance (0.5). The vertical dotted line corresponds to the data exclusion criterion used in the main analyses (150 nose pokes to the rewarding dispensers). For the main analyses only data to the right of this line were analysed and the purple and yellow data were pooled for each mouse, in order to calculate the discrimination performance for each condition. In most conditions the discrimination performance from the initial acquisition and reversal converges to similar values, indicating that the mice were sensitive to the reward properties and not only the location of the dispensers.

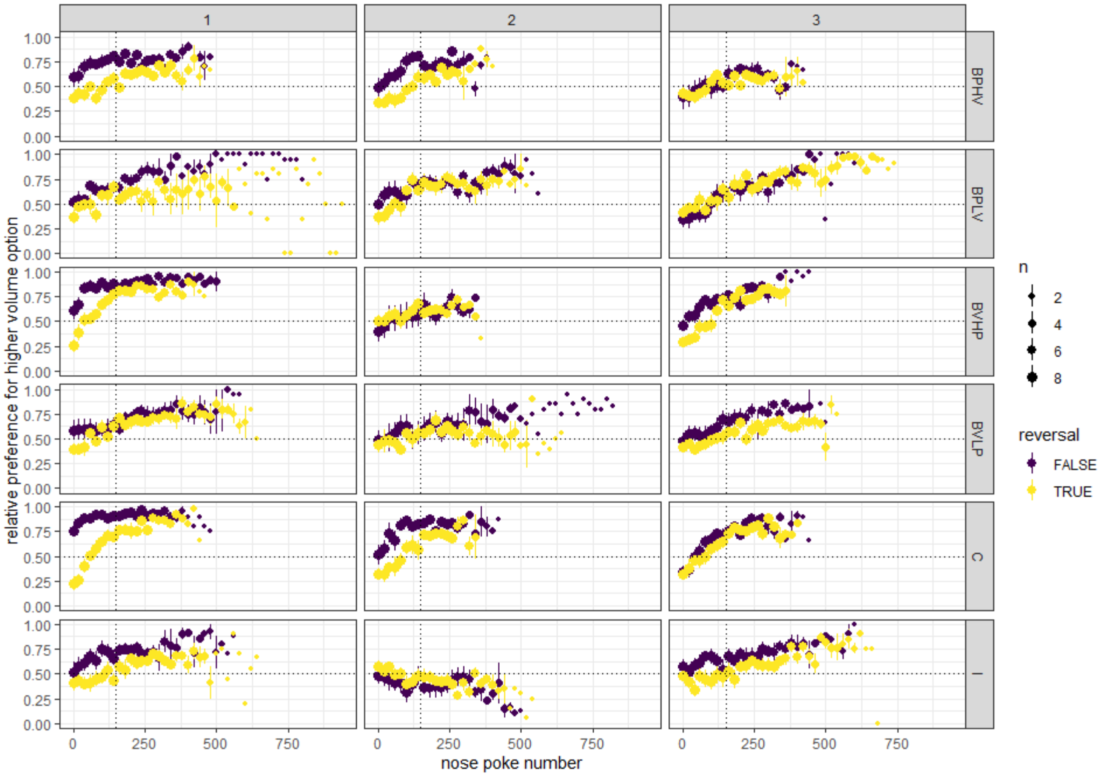

Figure S3. **Learning curves in experiment 2.** Same notation as in Fig. S2.

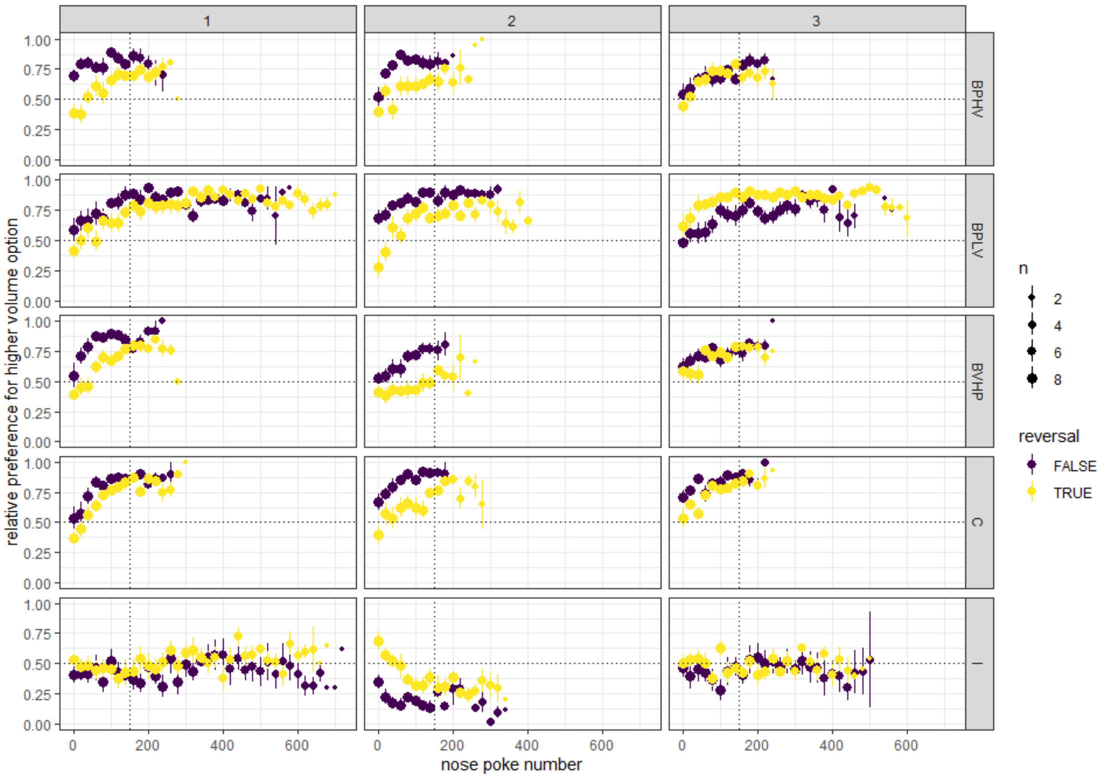

Figure S4. **Learning curves in experiment 3.** Same notation as in Fig. S2.

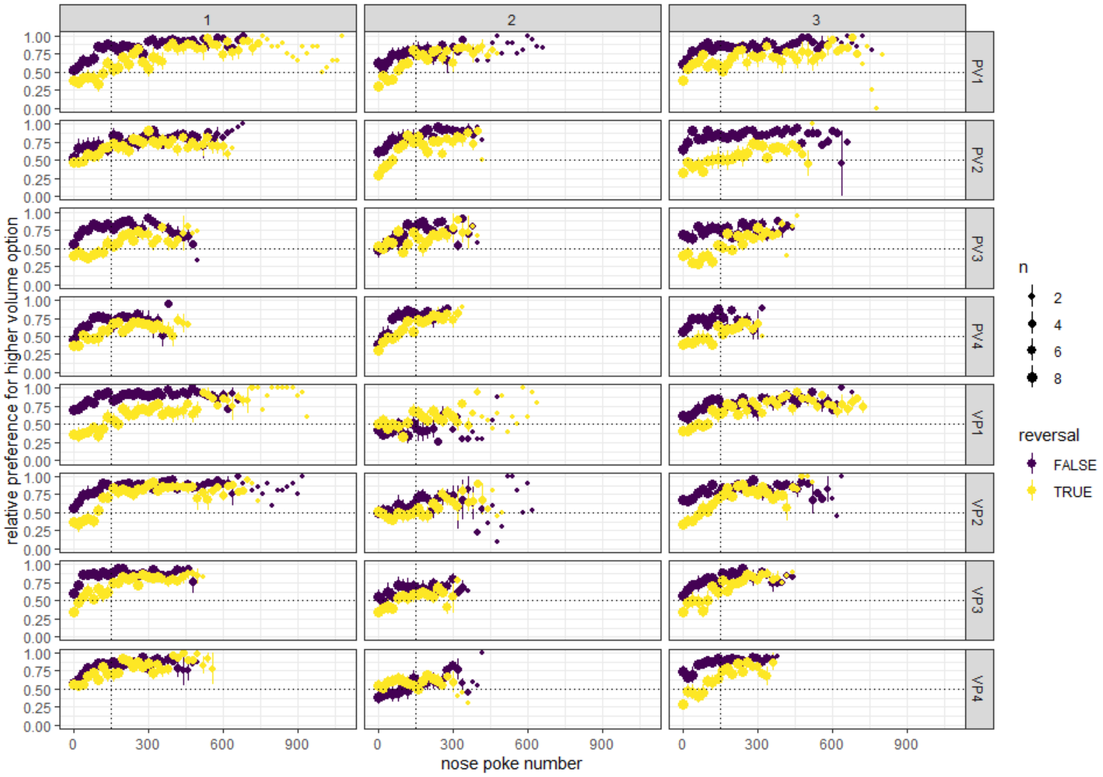

Figure S5. **Learning curves in experiment 4.** Same notation as in Fig. S2.

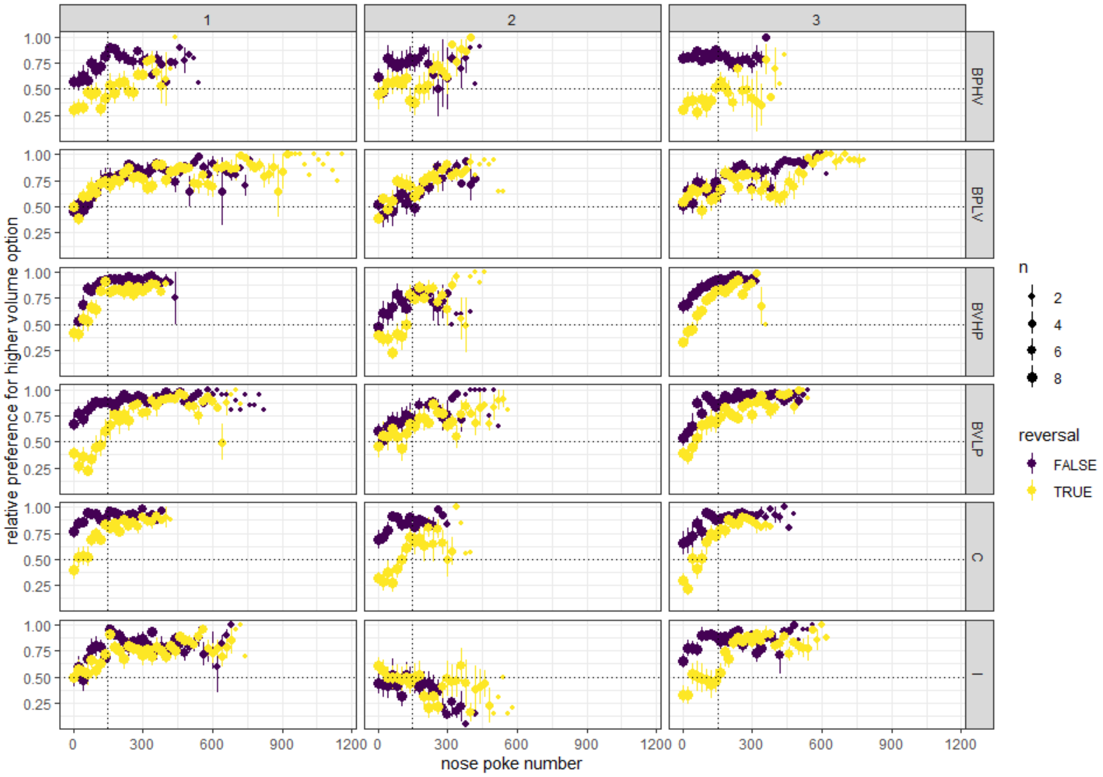

Figure S6. **Sensitivity tests for the models that only had γ as a free parameter.** Dots give the discrimination performances calculated from 1000 choices for each value of γ tested [0.05 , 2] and for each of the baseline conditions BPLV (purple) and BPHV (yellow). Lines give the corresponding fits based on locally weighted scatterplot smoothing (loess). The dashed line gives the empirical mean discrimination performance from the baseline conditions BPLV and BPHV and the green arrows point to the value of gamma that resulted in the smallest root-mean-square-errors (RMSEs). These values were then used in the main simulations (Table 1). The different panels give the results for the scalar expected value (**a**), two-scalar (**b**), and winner-takes-all (**c**) models.

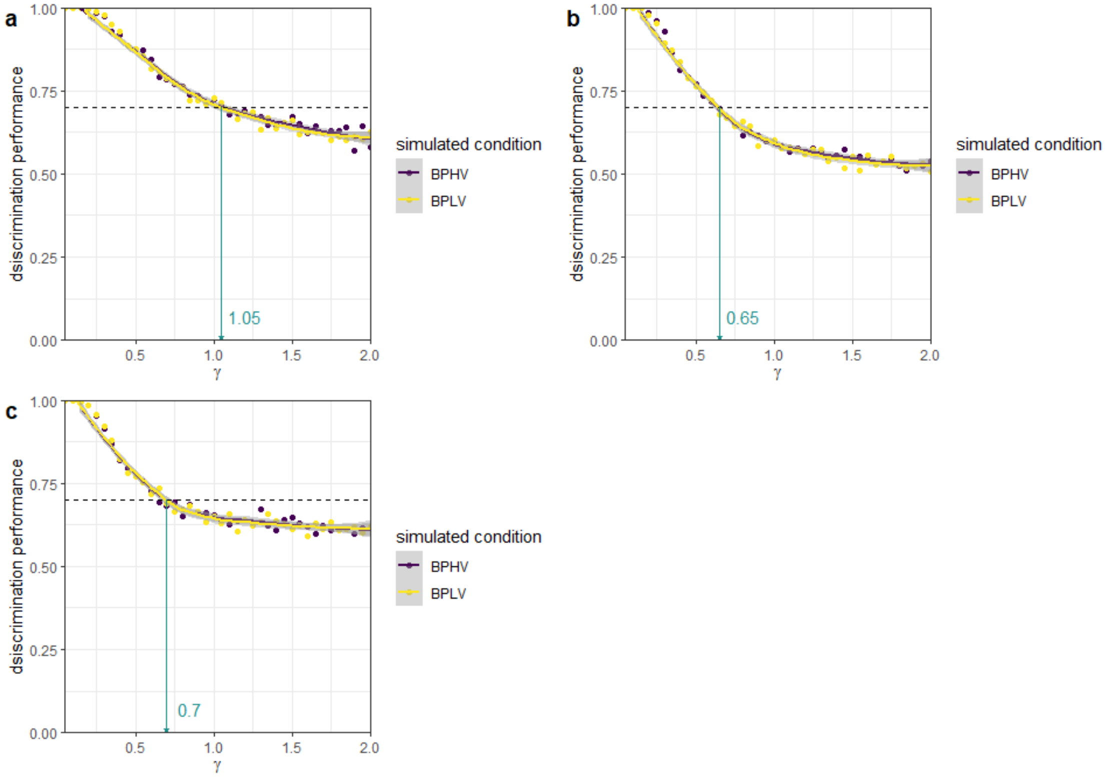

Figure S7. **Sensitivity tests for the randomly non-compensatory model.** Same notation as in Fig. S6. The different panels give the different values of the probability with which the volume dimension was chosen (θ_v). For a non-biased randomly non-compensatory model we set θ_v = 0.5.

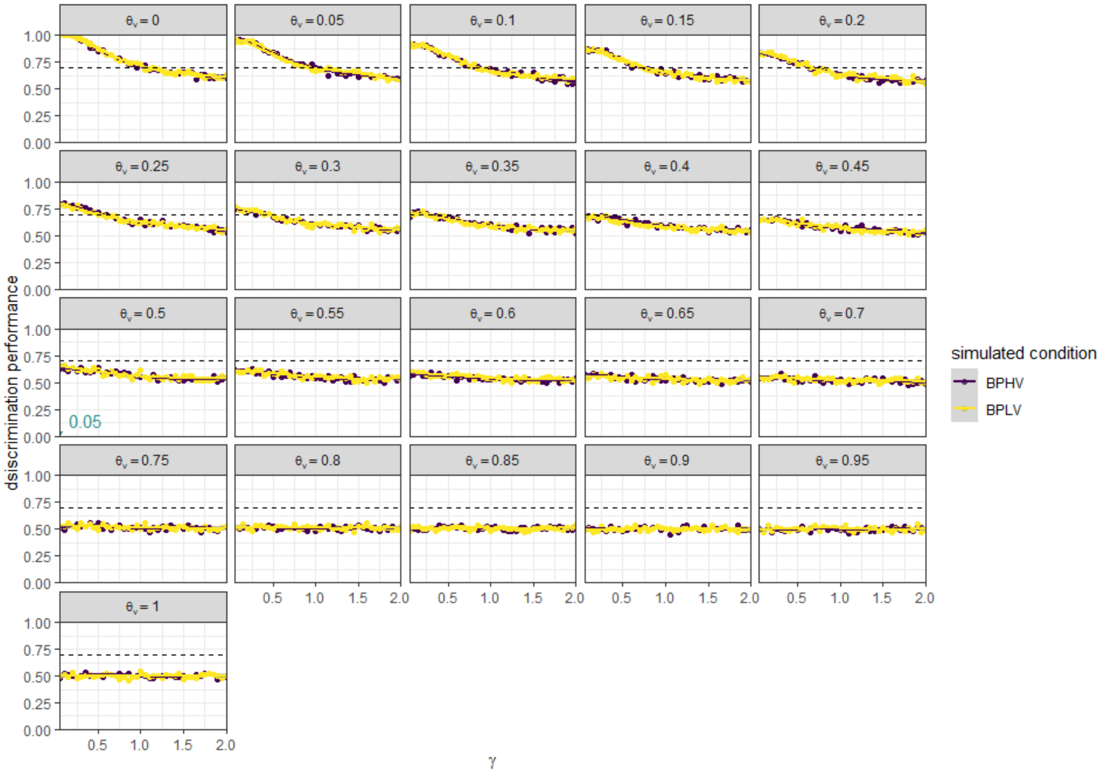

Figure S8. **Sensitivity tests for the probability first model.** Same notation as in Fig. S6. The different panels give the different values of the salience threshold that needed to be reached for one option to be preferred over the other. We set the value of the threshold for both the volume and probability dimensions to 0.8, based on the psychometric function threshold for probability (Rivalan, Winter, and Nachev 2017).

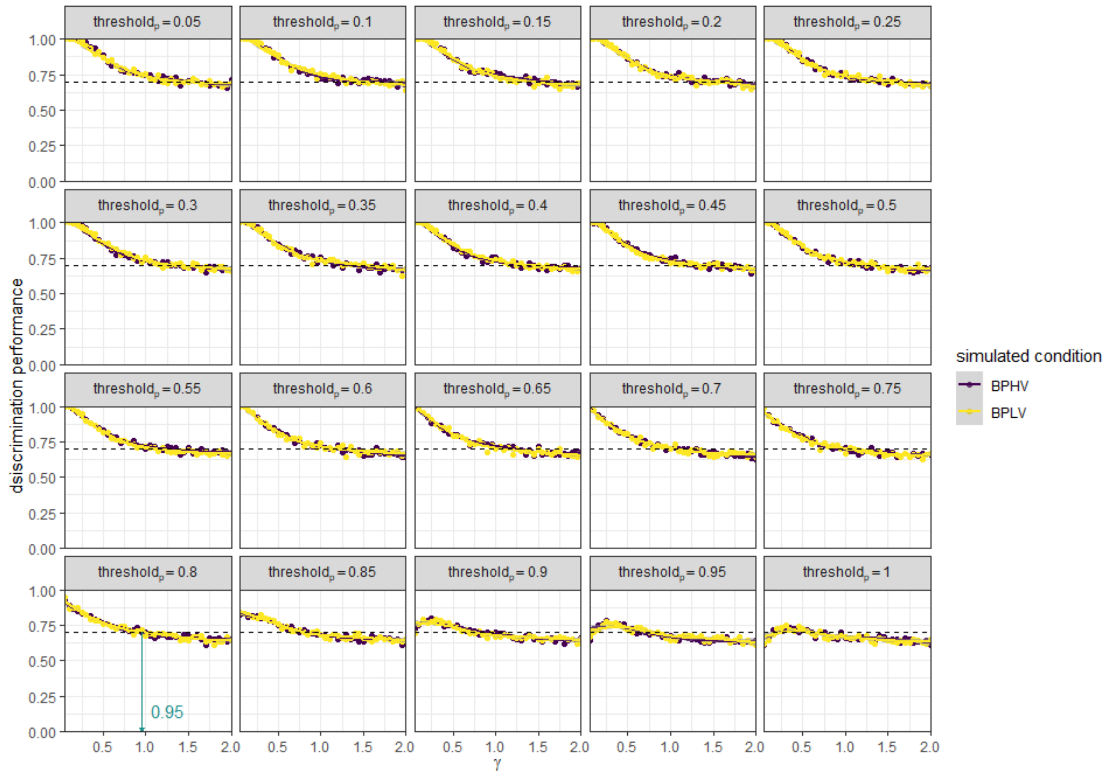

Figure S9. **Sensitivity tests for the volume first model.** Same notation as in Fig. S6. The different panels give the different values of the salience threshold that needed to be reached for one option to be preferred over the other. We set the value of the threshold for both the volume and probability dimensions to 0.8, based on the psychometric function threshold for probability (Rivalan, Winter, and Nachev 2017).

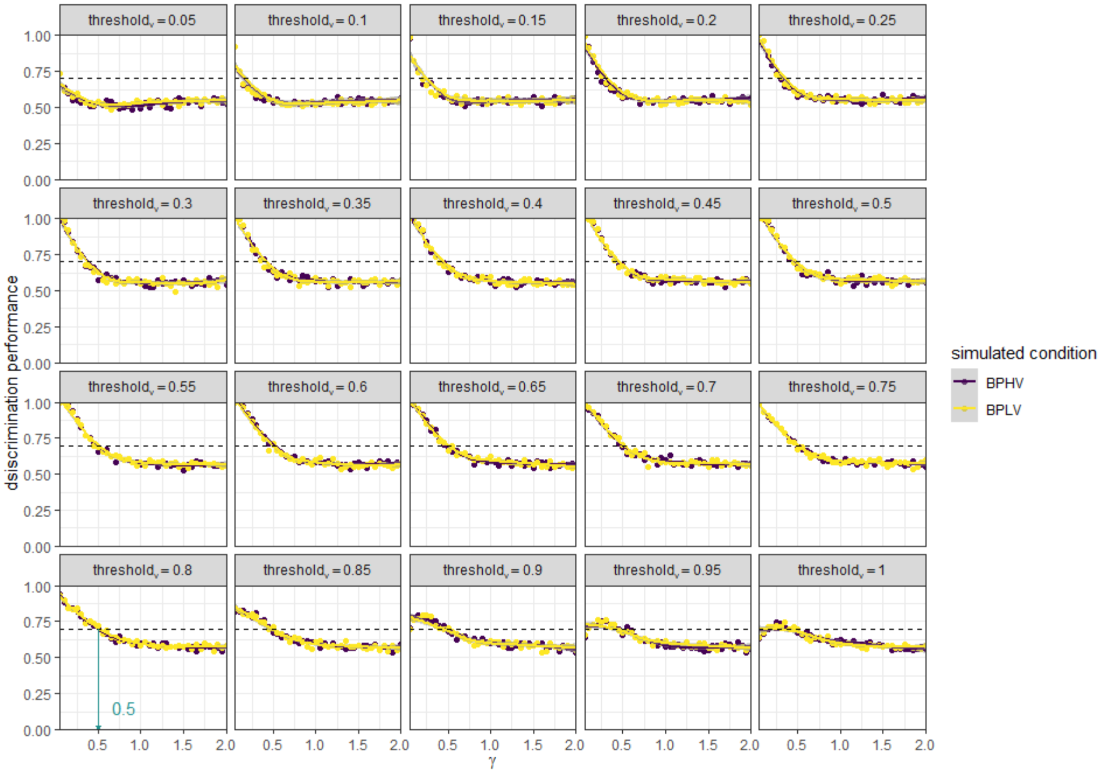

Figure S10. **Comparison of discrimination performance in all six simulation models and in the three mouse cohorts in Experiment 1**. Columns give the condition names (Fig. 2) and rows, the model number (Table 1). Empirical data from the three cohorts are represented by differently color-filled density curves from the observed discrimination performances. Simulation data are represented by an empty thick-lined density curve. The dashed line gives the median of the empirical data and the dotted line - the median of the simulated data. The discrimination performance gives the relative visitation rate of the more profitable option, or, in the incongruent condition, the option with the higher volume.

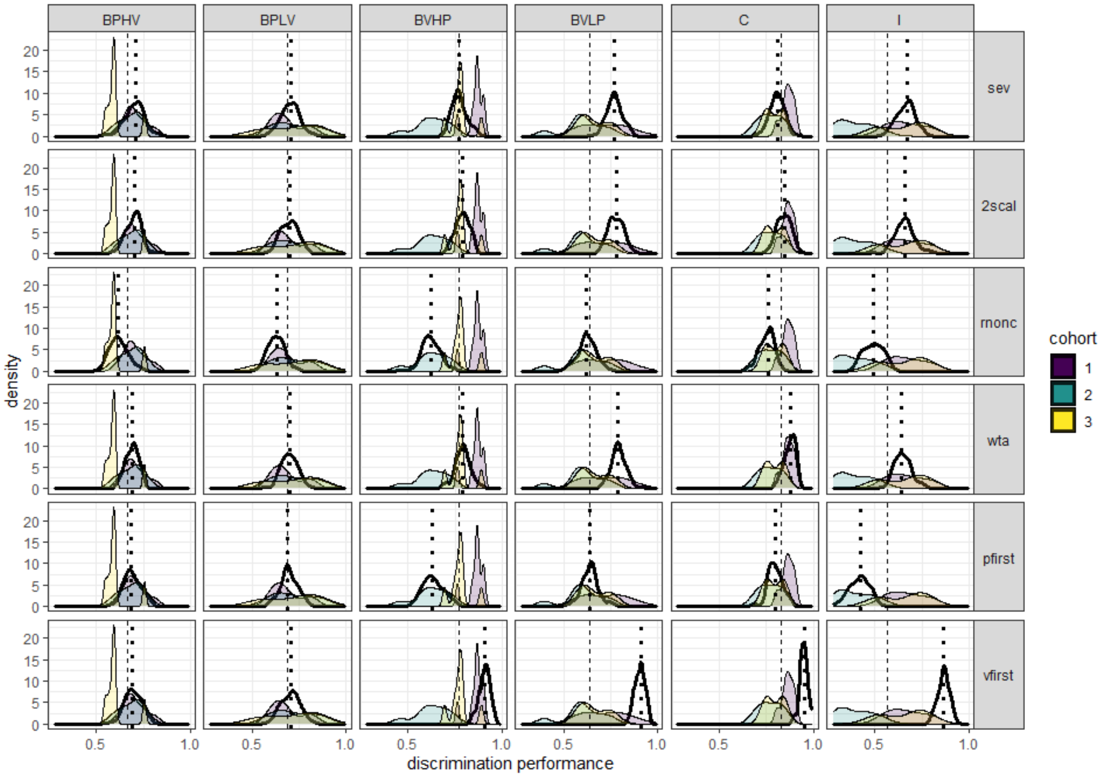

Figure S11. **Comparison of discrimination performance in all six simulation models and in the three mouse cohorts in Experiment 2**. Same notation as in Fig. S10.

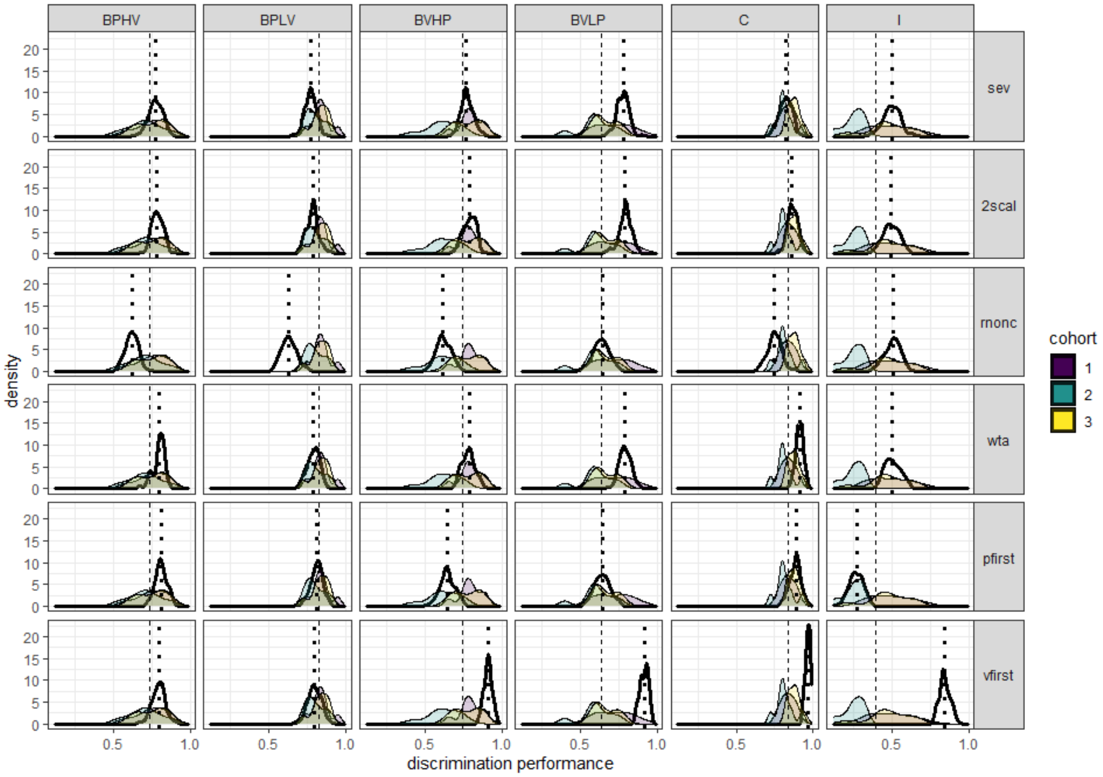

Figure S12. **Comparison of discrimination performance in all six simulation models and in the three mouse cohorts in Experiment 3**. Same notation as in Fig. S10.

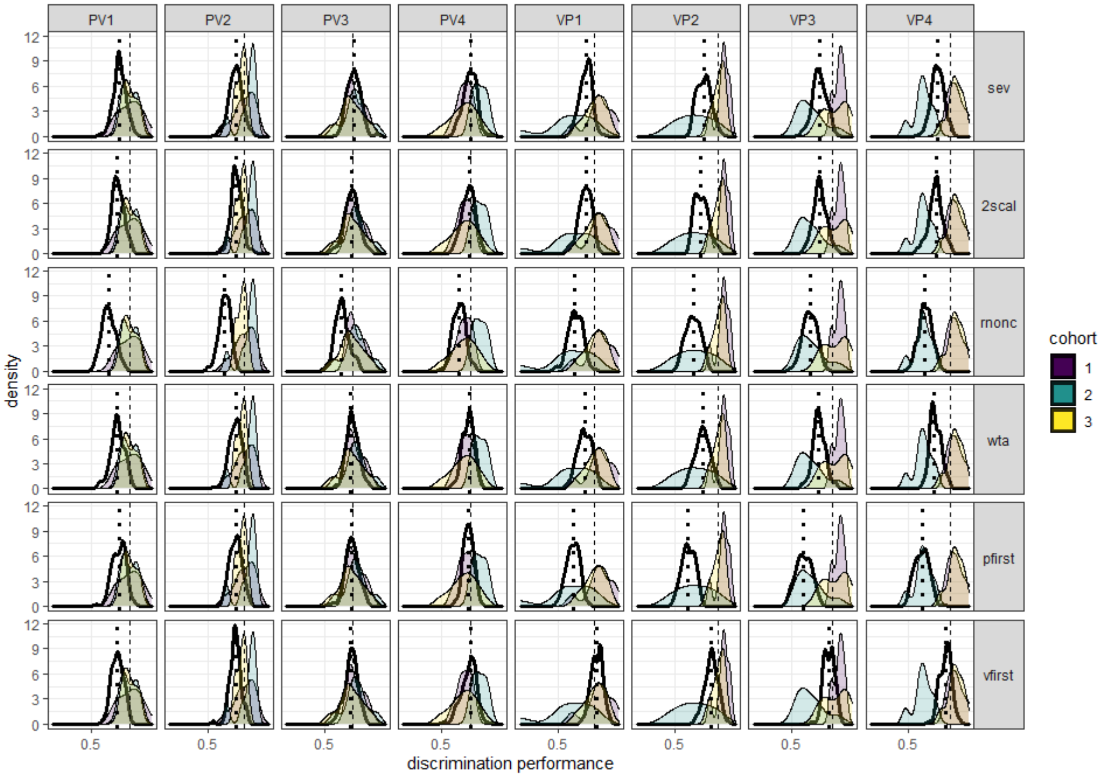

Figure S13. **Comparison of discrimination performance in all six simulation models and in the three mouse cohorts in Experiment 4**. Same notation as in Fig. S10.

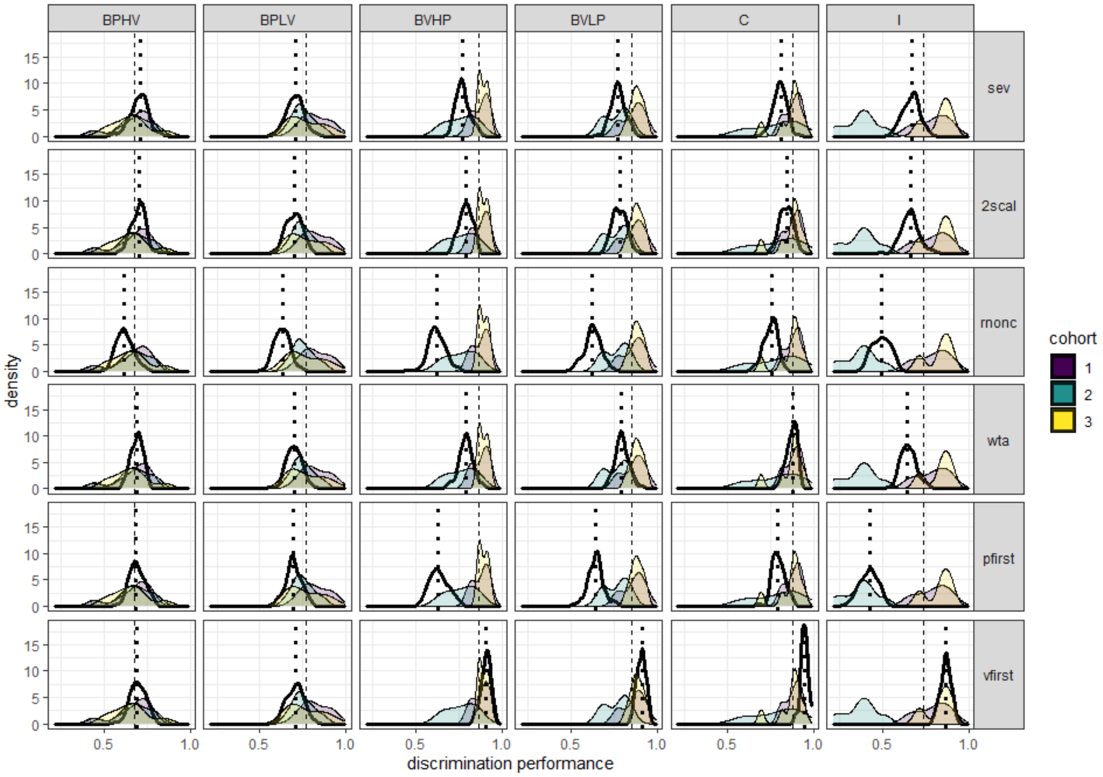

Figure S14. **Slope estimates for the effect of the background dimension on the discrimination performance in the relevant dimension for different decision models**. The two choice options always differed along the relevant dimension (either probability or volume) at a fixed relative intensity. The discrimination performance for 100 virtual mice making 100 decisions each was measured at four different levels of the background dimension. Symbols and whiskers give means and 98% confidence intervals estimated from bootstraps. The smallest effect size of interest (dashed lines) was determined to be the slope that would have resulted in a difference in discrimination performance of 0.1, from the lowest to the highest level of the background dimension. Compare to Fig. 5.

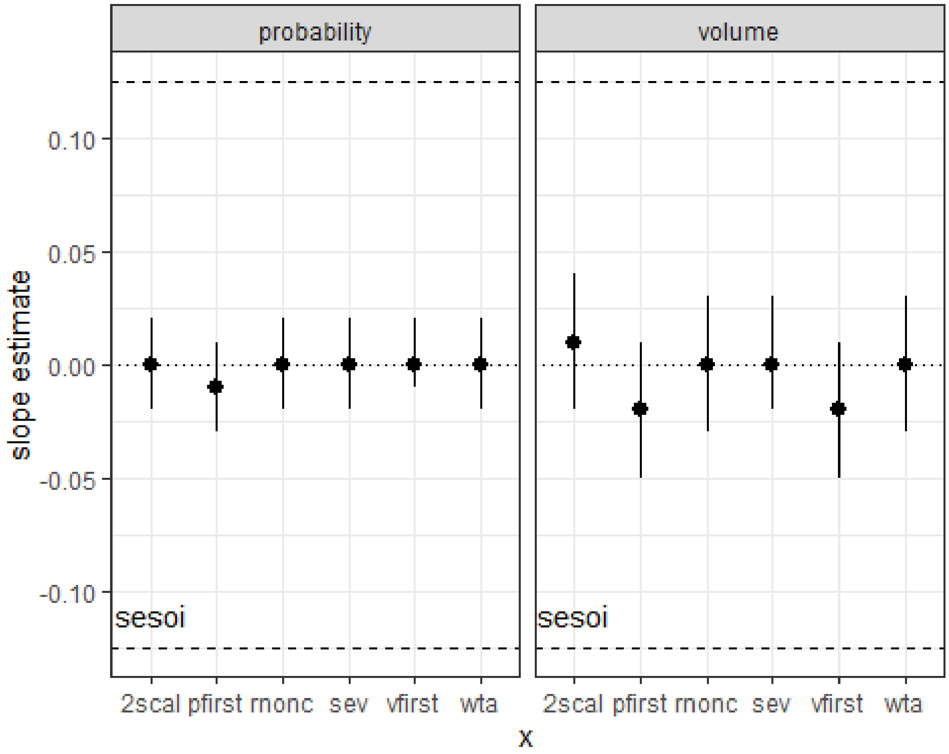

Figure S16. **Visit durations during rewarded and unrewarded nose pokes for the three cohorts in all experiments.** Columns give the status of the nose poke (rewarded or unrewarded) and rows, the experiment number (1-4). Data from the three cohorts are represented by differently color-filled density curves from the observed individual nose poke durations. Note the logarithmic scale on the abscissa.

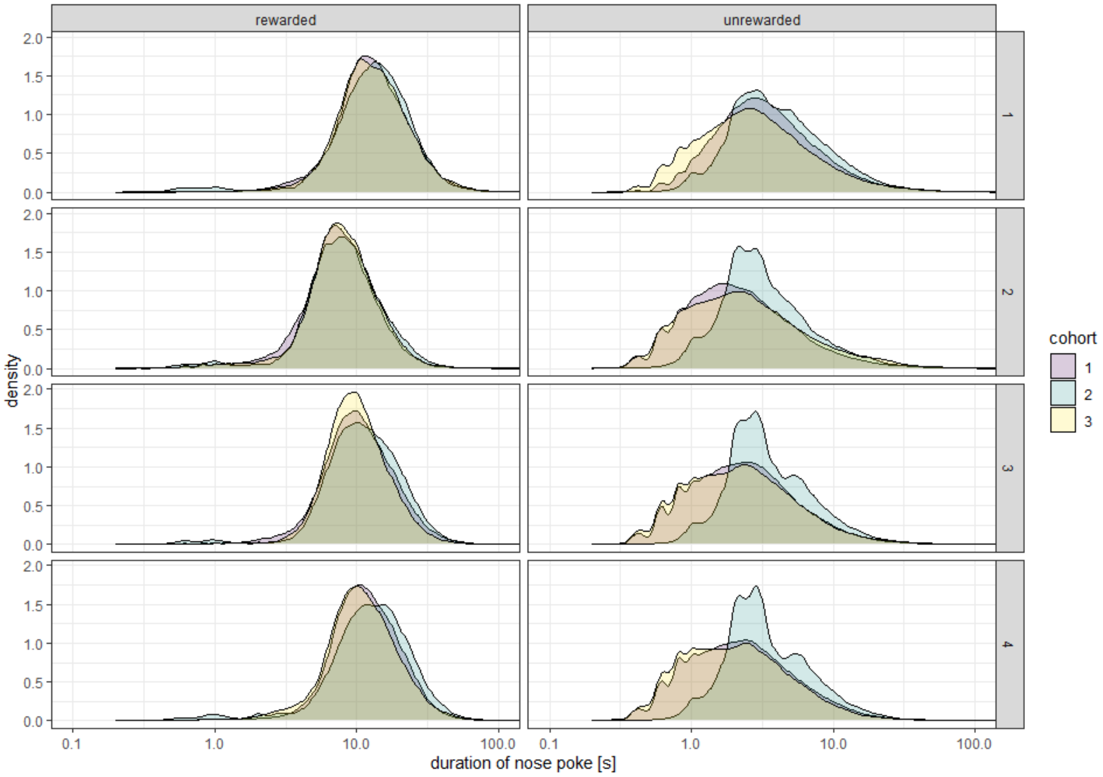

Supplement: Supplementary file 1 — Supplementary material 1 (pdf 832 KB) [file 10071_2021_1482_MOESM1_ESM.pdf]
